# The effect of melatonin on the mouse ameloblast-lineage cell line ALCs

**DOI:** 10.1038/s41598-022-11912-3

**Published:** 2022-05-17

**Authors:** Jing Pan, Qianhui Ren, Zhao Yang, Ying Guo, Kubin Kwon, Checheng Shen, Yueying Wang, Fang Ji

**Affiliations:** 1 Department of Orthodontics, Shanghai Ninth People’s Hospital, Shanghai Jiao Tong University School of Medicine, Shanghai, 200025 China; 2 State Key Laboratory of Medical Genomics, Shanghai Institute of Hematology, National Research Center for Translational Medicine at Shanghai, Rui Jin Hospital Affiliated to Shanghai Jiao Tong University School of Medicine, Shanghai, 200025 China

**Keywords:** Cell biology, Molecular biology, Medical research

## Abstract

Melatonin plays a critical role in promoting the proliferation of osteoblasts and the growth and development of dental papilla cells. However, the effect and mechanism of melatonin on the growth and development of ALCs still need to be explored. CCK8 assay was used for the evaluation of cell numbers. qRT-PCR was used to identify the differentially expressed genes in ALCs after melatonin treatment. The number and morphology of ALCs were investigated by confocal microscopy. Alkaline phosphatase assay and Alizarin red S staining were used for measuring mineralization. Then, we focused on observing the crucial factors of the signaling pathway by RNA-seq and qRT-PCR. Melatonin limited the cell number of ALCs in a dose-dependent manner and promoted the production of actin fibers. A high concentration of melatonin significantly promoted the mRNA levels of enamel matrix proteins and the formation of mineralized nodules. RNA-seq data showed that Wnt signaling pathway may be involved in the differentiation of ALCs under the influence of melatonin. This study suggests that melatonin plays a regulatory role in the cell number, differentiation, and mineralization of the ALCs, and then shows the relationship between the Wnt signaling pathway with the ALCs under melatonin.

## Introduction

Amelogenesis imperfecta (AI), a heterogeneous group of genetic dental rare diseases cause patients symptoms of eating difficulties and feeling pain, which is difficult to treat^1^. AI includes deficiencies in the amount of enamel and a hypoplastic, and/or hypomineralized enamel phenotype, which is caused by mutations in numerous genes^2^. For example, amelogenin X-linked (AMELX) contributing ~ 90% of the enamel matrix proteins (EMPs), is essential for enamel crystallite growth and prismatic or rod architecture on the microscale^3^. It is known that there are many allelic mutations in the *AMELX* gene causing different alterations in the protein and resulting in different phenotypes^4^. AMELX alone is a weak promoter of nucleation but the addition of enamelin (ENAM) promotes nucleation rates in a highly nonlinear, nonmonotonic manner, which reaches a sharp maximum at a ratio of 1:50 ENAM/AMELX^5^. Possibly only isolated ENAM but not ENAM oligomers can act as sites of enhanced nucleation^6^. Mutations in the *ENAM* gene result in generalized thin enamel hypoplasia or bands of pitted enamel^2^. Odontogenic, ameloblast associated (ODAM) is considered one of the regulators of enamel mineralization and crystal elongation^7^, and its expression in the dental junctional epithelium helps maintain the integrity of the junctional epithelium attachment^8,9^. As above, enamel formation is a highly regulated process, which may be affected by various negative effects^2^. Therefore, it is urgent and necessary to study the influencing factors of enamel formation.

Melatonin (MT) is mainly secreted by the pineal gland (PT) in the brain and is regulated by the suprachiasmatic nucleus (SCN). It is one of the neuroendogenous hormones affecting the biological cycle^10,11^. It not only regulates the periodic rhythm, body temperature^12^, and blood pressure^13^, but also plays a role as an anti-cancer agent^14^, and regulates endocrine system activities^15^. Melatonin produces a marked effect by activating the high-affinity melatonin receptor (MTNR) in target tissues. MTNR1A and MTNR1B are two subtypes of MTNR in humans and mammals.

In recent years, the biological role of melatonin in the oral cavity has been widely concerned^16,17^. Melatonin increases the stability of implant-bone osseointegration, strengthen the density of bone area, and reduces alveolar bone loss^18^. Meanwhile, melatonin promoted the differentiation of dental papilla cells and the expression of dentin sialoprotein (DSP)^19^, and induced the osteogenic differentiation of dental pulp mesenchymal stem cells^20,21^. According to the research report, MTNR is expressed in human tooth germ and mouse tooth epithelial cell line HAT-7^22^. When the SCN with high melatonin receptor expression in experimental animals was removed, the circadian growth and development of teeth immediately disappeared^23^. Therefore, we put forward the hypothesis for the first time that the rhythm change of melatonin and its receptor may be one of the mechanisms of tooth dysplasia^24^. To test the hypothesis, we found that after changing the circadian rhythm of BALB/c pregnant mice, the protein levels of MTNR1A and MTNR1B in neonatal mouse tooth germs changed in varying degrees. We injected a melatonin receptor antagonist into the peritoneum of BALB/c pregnant mice. It was found that the ameloblasts of mice at 7 and 10 days after birth were widely denatured, the degree of enamel mineralization was low, and the content of AMELX was significantly reduced^25^. The previous experimental results showed that melatonin and its receptor system were related to the periodic growth and development of enamel. However, we have not conducted in vitro experiments on melatonin-treated ameloblasts.

The ameloblast-lineage cell line (ALCs) was initially obtained from the organ of mandibular molar tooth germ of newborn (C57BL/6 J) mice by Akira Nakata et al.^26^. ALCs are a spontaneously immortalized ameloblast cell line and are thought to be in the early stages of maturation. They consist of enamel epithelioid structure formation, present ameloblast characteristics, and express specific enamel genes, such as Amelx, Enam and Odam. In addition, ALCs in the standard medium could form calcified nodules^26,27^.Thus, ALCs were proved to be a mature tool to study melatonin-medicated effect.

To explore the influencing factors of enamel development from the cellular level and the possible factors of enamel hypoplasia, we studied the effects of melatonin on the proliferation, differentiation, and mineralization in ALCs in vitro. The effects of Luzindole, a melatonin receptor antagonist, on ALCs were also studied. Then, we focused on observing the crucial factors of the signaling pathway.

## Results

### Effect of melatonin on the proliferation of ALCs

**Figure 1:**
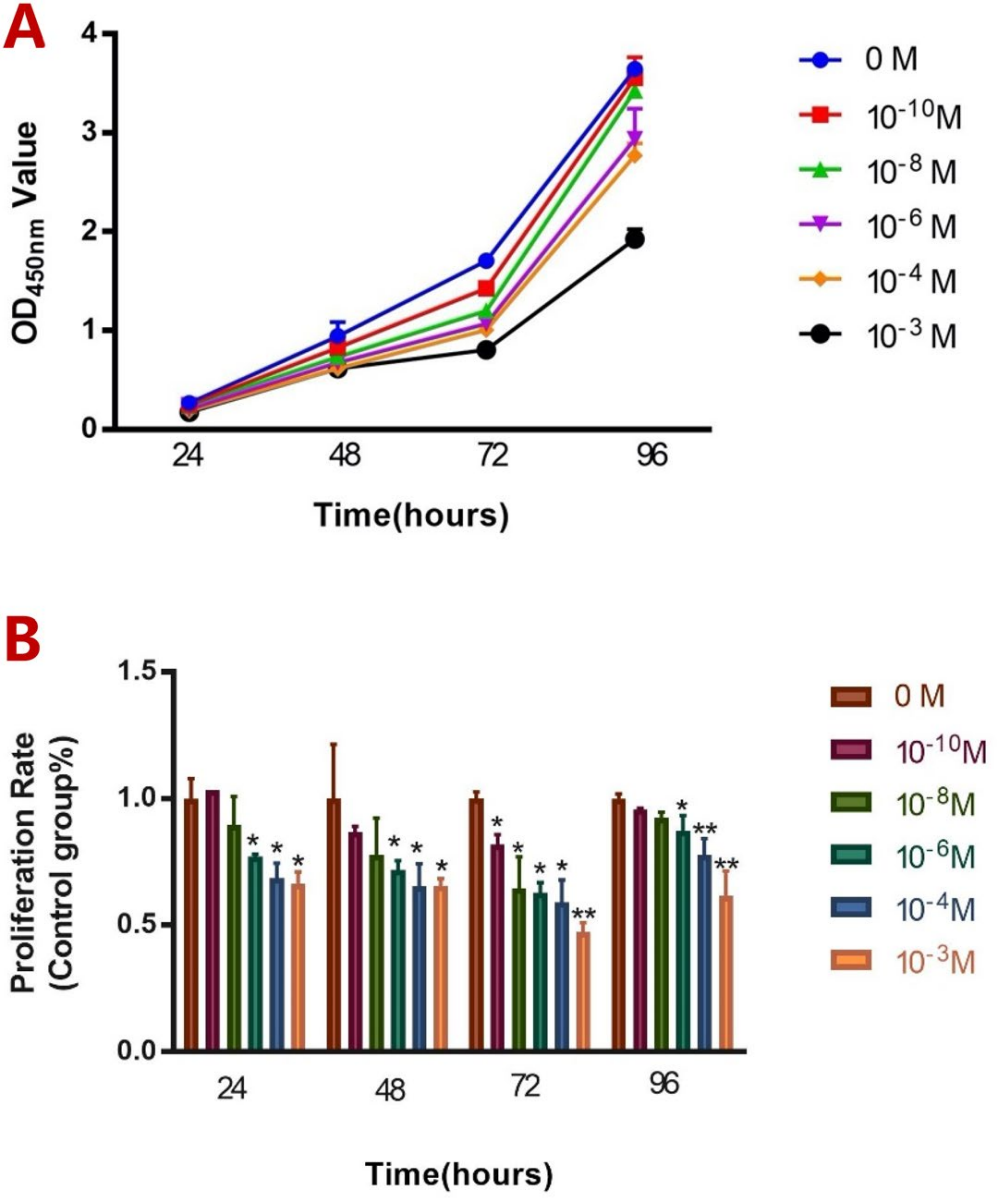
Effect of melatonin on ALCs’ cell number. Cells were treated with various concentrations of MT (0, 10^–10^, 10^–8^, 10^–6^, 10^–4^ and 10^−3^ M) for 24, 48, 72 and 96 h respectively. Subsequently, the viability of the cells was measured via a CCK8 assay. Each value was presented as mean ± SD for triplicate cultures. (**A**) Growth curve of ALCs in different concentrations of MT at different periods. (**B**) The proliferation rate of ALCs in different concentrations of MT at different periods. (*p < 0.05, **P < 0.001, compared to control).

To evaluate the effect of melatonin on the cell number of ALCs, concentration gradient of 0, 10^–10^, 10^–8^, 10^–6^, 10^–4^ and10^−3^ M were set up, and the absorbance value at 450 nm was detected by the CCK-8 method. Melatonin inhibited the increase in ALCs’ cell number in a time-dependent (Fig. 1A) and dose-dependent manner (Fig. 1B).

### Effect of melatonin on the morphology of ALCs

**Figure 2:**
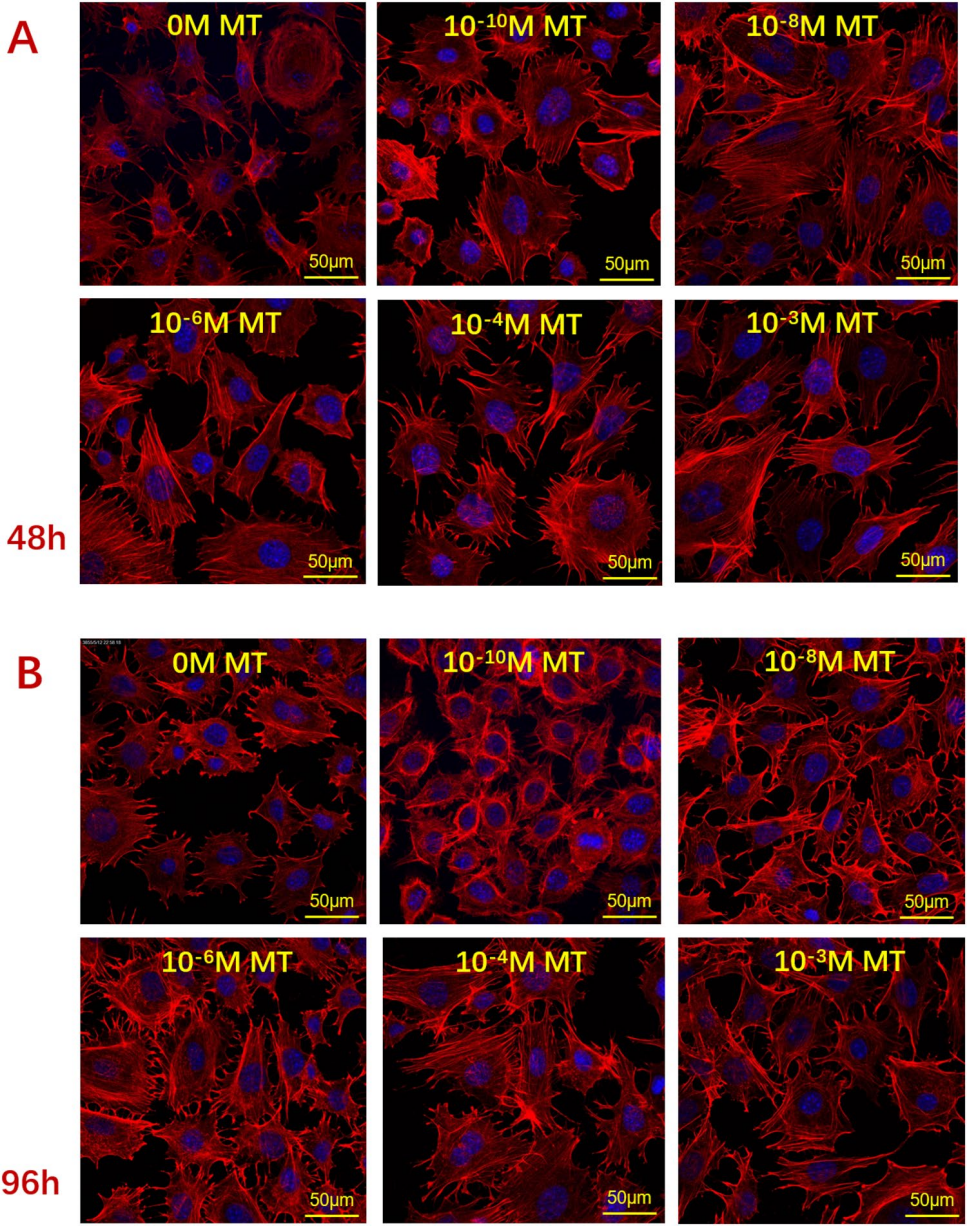
Effect of melatonin on ALCs’ morphology. Cells were treated with various MT concentrations (0, 10^–10^, 10^–8^, 10^–6^, 10^–4^, and10^−3^ M) for 48 and 96 h, respectively. Subsequently, the cells were observed by laser scanning confocal microscope (LSCM). The magnification is 50 μm. Cells were treated with MT concentrations for 48 h (**A**) and 96 h (**B**), respectively.

The morphology of ALCs was investigated via rhodamine-labelled phalloidin/DAPI staining and confocal image analysis. After 48 h of melatonin treatment, the changes in cell number and myofilament protein in the experimental group were not noticeable compared with the control group. However, ALCs morphology differed remarkably between melatonin and standard medium after 96 h. At the 96 h time point, cells in melatonin showed plenty of pseudopods and more red fluorescence of actin filaments than those in the standard medium. With the increase in melatonin concentration, cell numbers were significantly decreased (Fig. 2).

### Effect of melatonin on the differentiation of ALCs

**Figure 3:**
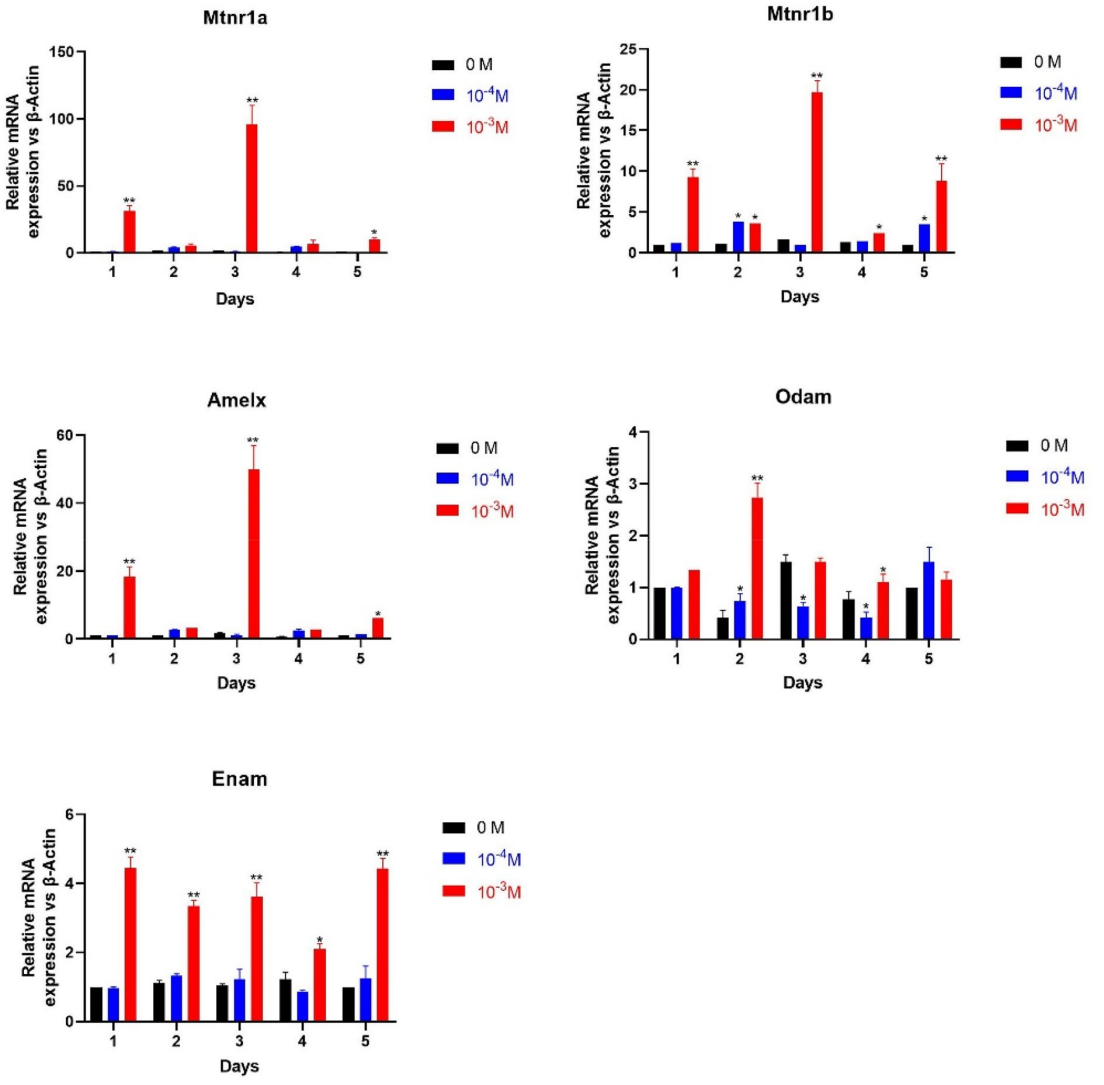
Effect of melatonin on expression of specific enamel genes in ALCs. Cells were treated with various concentrations of MT (0, 10^–4^ and10^−3^ M) for 1, 2, 3, 4, and 5d. Subsequently, the expression of specific enamel genes and MTNRs was measured in qRT-PCR. (*p < 0.05, **P < 0.001, compared to control).

qRT-PCR was used to analyze the mRNA levels of EMPs in different concentrations of melatonin (Fig. 3). In the previous experiment, we explored the optimal concentration of melatonin on ALCs’ differentiation at gradient of 10^–3^, 10^–4^, 10^–6^, 10^–8^, 10^−10^ M, and found that 10^−4^ M and 10^−3^ M were suitable for the study of melatonin in ALCs. Therefore, 10^−4^ M and 10^−3^ M were selected for further study. In the experimental group, 10^−4^ M melatonin concentration had no significant effect on the specific enamel genes and MTNRs after 5 days. However, the expression of Mtnr1a and Mtnr1b in 10^−3^ M melatonin group showed an evident and similar change trend, both of which reached the peak on the third day. The expression of MTNRs was 70 times and 20 times higher than that of the control group (0 M MT), respectively. Amelx, Enam and Odam expression levels increased significantly in cells cultured in the medium containing 10^−3^ M melatonin (P < 0.05). The expression of Amelx and MTNRs showed the similar trend, reaching a peak on the third day, and the expression level of Amelx was 50 times higher than that of the control group. The expression levels of Enam and Odam were 5 times and 1–2 times higher than those in cells in the standard medium, but the changing trend was different from that of MTNRs. As a result, melatonin has been shown to increase the mRNA expression of these specific enamel genes.

### Effect of melatonin on the mineralization of ALCs

**Figure 4:**
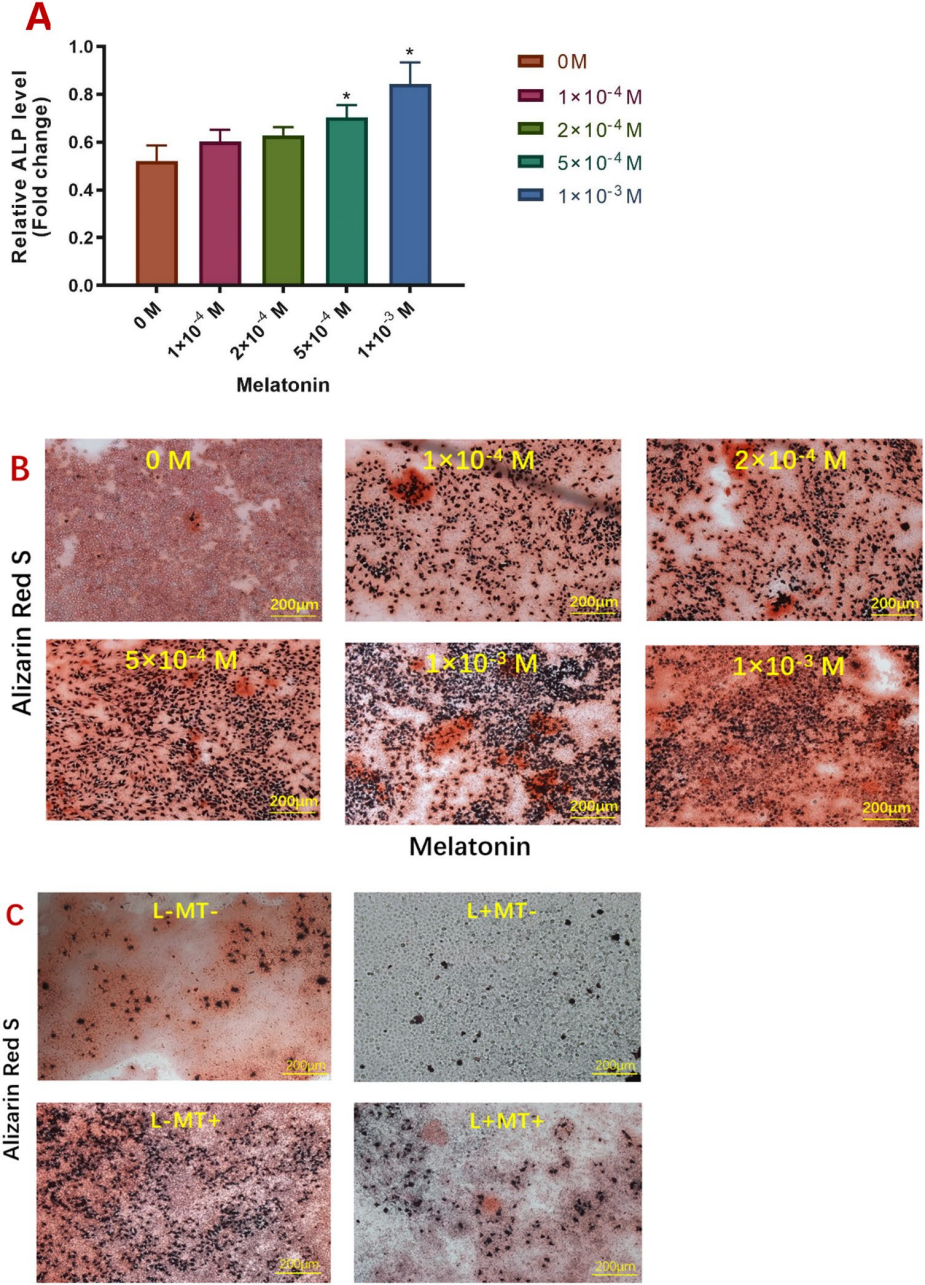
Effect of melatonin on ALCs’ mineralization. (**A**) ALP activity in ALCs under MT treatments (0, 1 × 10^–4^, 2 × 10^–4^, 5 × 10^–4^, 1 × 10^−3^ M) was determined on day 7. Each value was presented as mean ± SD for triplicate experiments. (*p < 0.05, **P < 0.001, compared to control). The magnification is 200 μm. (**B**) Effect of melatonin on the formation of the mineralized nodule in ALCs. Cells were treated with various concentrations of MT (0, 1 × 10^–4^, 2 × 10^–4^, 5 × 10^–4^, 1 × 10^−3^ M) for 28 days. (**C**) Effect of melatonin and Luzindole on the formation of mineralized nodules of ALCs. Cells were incubated with the conditioned medium ((L-MT−), (L + MT−), (L-MT+), (L + MT+)) for 28 days.

Alkaline phosphatase (ALP) activity was used to evaluate the mineralization of ALCs in different concentrations of melatonin medium (0, 1 × 10^–4^, 2 × 10^–4^, 5 × 10^–4^, 1 × 10^−3^ M). The ALP activity of ALCs in the 1 × 10^−3^ M and 5 × 10^−4^ M melatonin mediums was significantly higher than that in the standard medium (P < 0.05). With the increase in melatonin concentration, the ALP activity of ALCs gradually increased, indicating an enhancement of mineralization potential (Fig. 4A).

Alizarin red staining was used to evaluate mineralized nodule formation in ALCs exposed to different concentrations of melatonin medium and melatonin receptor antagonist. Only a few calcified nodules were stained in the control group (0 M MT). There were more calcified nodules in the 1 × 10^–4^, 2 × 10^–4^ and 5 × 10^−4^ M melatonin groups. After repeated experiments, dense and obvious calcified nodules were produced in the 1 × 10^−3^ M melatonin medium. Compared with the control and low-concentration melatonin groups, the red staining became darker, and the number of calcified nodules increased significantly in this medium. With the increase in melatonin concentration, calcified nodules of ALCs were dense and obvious (Fig. 4B).

In the other half of the samples, ALCs in standard medium (L-MT−) produced a small amount of calcification. Those in medium supplemented with Luzindole (L + MT−) had no apparent calcified nodules, with the alizarin red staining yielding negative results. The calcified nodules of melatonin in the absence of Luzindole (L-MT+) were significantly increased and dense. With the presence of Luzindole (L + MT+), calcified nodules decreased, and alizarin red staining was weakly deepened, which suggested that the mineralization of ALCs depends on melatonin receptor Mtnr1a and Mtnr1b. The results showed that melatonin promoted the mineralization of ALCs, and Luzindole inhibited the mineralization of ALCs (Fig. 4C).

### Signaling pathway of melatonin in ALCs

**Figure 5:**
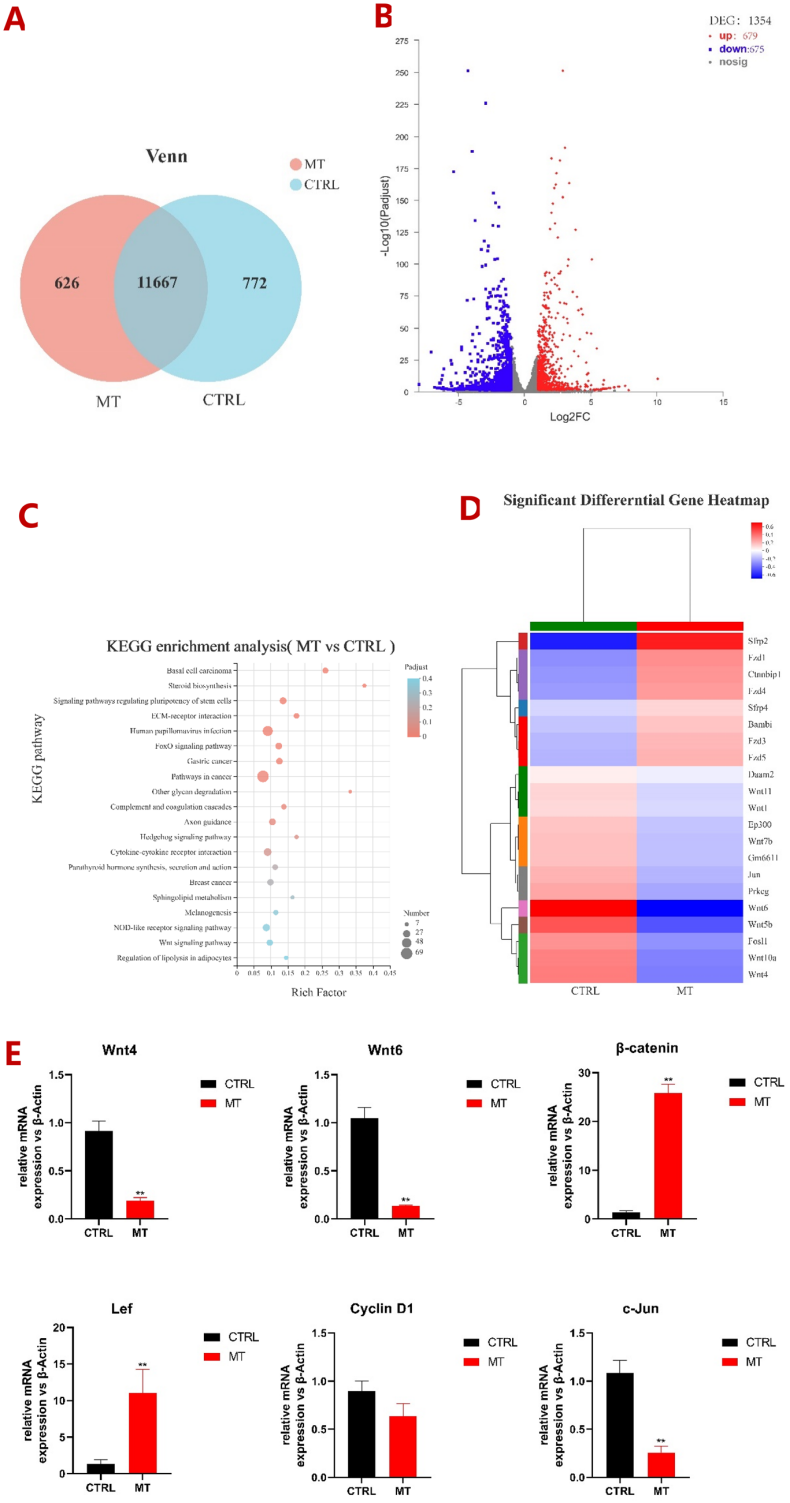
RNA sequencing Analysis. MT: melatonin, CTRL: control. (**A**) Venn diagram revealed unique and overlapping gene expression between CTRL and MEL. (**B**) Volcano plots revealed significant changes in [MT (n = 2) relative to CTRL (n = 2)] genes in ALCs. Each dot in the volcano plots represents a detected gene. In the MT group, the genes with significantly increased [fold change > 2, P < 0.001] were shown in red. While those in CTRL group significantly decreased [fold change < 2, P < 0.001] were shown in blue. (**C**) KEGG enrichment analysis of DEGs in ALCs (Only the top 20 KEGG signaling pathways were selectively extracted). The vertical axis indicates the enrichment of KEGG signaling pathways. (**D**) The heat map of Wnt signaling pathway-related gene expression in the control group and melatonin treated ALCs for 3d. (**E**) The main factors of the Wnt canonical pathway in ALCs treated with/without melatonin for 3d were verified by qRT-PCR.

Since we found that 10^−3^ M melatonin exerted the most obvious impact on gene expression in ALCs after 3-day melatonin treatment, which was confirmed by qRT-PCR, we then performed high-throughput transcriptome sequencing of ALCs treated with or without 3-day melatonin treatment to reveal the mechanism of melatonin-medicated gene expression. The Venn diagram (Fig. 5A) showed 11,667 overlapping expressed genes between two groups, 626 differentially expressed genes in melatonin treatment (MT) group, and 772 differentially expressed genes in control (CTRL) group, respectively. After comparing the two groups according to the requirements of “Fold Change ≥ 2, significance level P < 0.001”, the volcano map showed a total of 1354 differentially expressed genes (DEGs) were identified, of which there were 679 up-regulated genes and 675 down-regulated genes (Fig. 5B). These results indicated significant alterations in gene expression during differentiation of ALCs after melatonin treatment. However, we found the expression of Amelx and Enam was very low in ALCs, below the detection level when analyzed by RNA-seq. KEGG pathway enrichment analysis^28^ showed 20 statistically significantly enriched DEG pathways (Fig. 5C). DEGs were largely enriched in the FoxO signaling pathway, Hedgehog signaling pathway, Wnt signaling pathway, NOD-like receptor signaling pathway and signaling pathways regulating pluripotency of stem cells.

The Wnt signaling pathway, closely related to tooth growth and development, attracted our attention. According to the results of RNA-seq, melatonin induced the expression of Wnt signaling pathway factors in ALCs at least twice as much as that in the control group (Fig. 5D). These genes were Wnt genes (Wnt1, Wnt4, Wnt5b, Wnt6, Wnt7b, Wnt10a and Wnt11), Frizzled family genes (Fzd1, Fzd3, Fzd4 and Fzd5), secreted frizzled-related genes (Sfrp2 and Sfrp4), and the cAMP response element-binding protein (CBP)-encoding gene. Among them, the expression of Sfrp2, Sfrp4, Fzd1, Fzd3, Fzd4 and Fzd5 genes in the melatonin group increased significantly, while that of Wnt4, Wnt5b, Wnt6, Wnt7b and Wnt10a genes decreased significantly.

The regulation of the Wnt signaling pathway factors was confirmed by qRT-PCR analysis (Fig. 5E), including the Wnt genes, conduction factor β-catenin, the transcription factor Lef, and the downstream factors c-Jun N-terminal kinase (c-Jun) and Cyclin D1. Compared with the control group, the expression levels of Wnt4 and Wnt6 in 10^−3^ M melatonin were significantly decreased, which was consistent with the results of RNA-seq. Although the expression of β-catenin and Lef did not change significantly in RNA-seq, we also detected the expression of β-catenin and Lef because they are critical factors in the Wnt canonical pathway. However, the expression of β-catenin and Lef increased significantly in the experimental group, 27 times and 12 times higher, respectively. The downstream factor Cyclin D1 had no significant difference in expression level, while the expression level of the c-Jun gene was approximately 2 times that of the control group. The change in expression of these factors confirmed that the critical factors of the Wnt/β-catenin signaling pathway might play a role in the effect of melatonin-treated ALCs.

## Discussion

AI is common in oral clinic, which usually causes the incidence of oral diseases such as caries and premature tooth loss and even seriously affect the self-esteem of patients^29^. Enamel development is a highly regulated process, which can be perturbed by many environmental conditions and genetic alterations^2^. It is of great clinical significance to further study the impacting factors in enamel development during which the pathological changes may cause or boost AI. In the field of molecular developmental biology, many literatures on melatonin in oral hard tissue have reported that melatonin is referred to tooth growth and development, such as regulating the cellular function of odontogenic cells^20^, affecting tooth regeneration by MT-induced DNA methylation machinery^30^. However, the effect of melatonin on ameloblasts remains unclear. This study aimed to research on the effect of melatonin on ameloblasts and explore the possible mechanisms.

Melatonin has been shown to modulate the proliferation of various cells effectively^31^. For example, 1 mM melatonin inhibits the proliferation of breast cancer MCF-7^32^ and umbilical vein endothelial cells^33^ in vitro. Melatonin (10^–6^ to 10^−4^ M) has no noticeable effect on the proliferation of human osteoblast cell line hFOB1.19^34^, but melatonin (10^–12^ to 10^−10^ M) has an apparent inhibitory effect on the proliferation of human dental pulp cells or HDPCs^35^. All the evidence suggests that melatonin may have different sensitivity and specificity in inhibiting cell proliferation in vitro. Our study strongly confirmed the evidence that melatonin concentration of 10^−3^ M inhibited the number of ALCs in a time- and concentration-dependent manner. Then, we found that ALCs were more evenly distributed and produced more pseudopodia in the melatonin medium than in the standard medium. To some extent, with the increase in melatonin concentration, myofilament proteins were more densely arranged and interwoven, but the number of cells had decreased simultaneously, which was similar to the results of the cell proliferation experiment. The changes in cell number and myofilament protein levels were not apparent at 48 h, compared with 96 h. Therefore, we speculated that melatonin may promote actin fiber production and increase cytoskeleton construction time- and concentration-dependent.

In our previous work, loss of circadian rhythm mainly inhibited enamel formation and Amelx expression in mice through the melatonin receptor^25^. Therefore, we hypothesized that melatonin might promote the amelogenic behavior of ALCs. According to the experimental results, melatonin receptors Mtnr1a and Mtnr1b are present and stably expressed in ALCs, suggesting that ameloblasts may be one of the targets of melatonin. We found that 10^−3^ M melatonin significantly enhanced the expression of specific enamel genes in ALCs. The expression curve of Amelx mRNA was consistent with that of Mtnr1a and Mtnr1b, which peaked on the third day and then declined in the following days, suggesting the expression of Amelx in melatonin group may be associated with MTNRs. Melatonin stimulates the differentiation of enamel cells, which is consistent with previous experimental results in vivo. However, the expression curve of Enam and Odam was relatively stable within 5d. There was a significant difference compared with the control group, and it did not change with the melatonin receptor expression. This suggests that melatonin also promotes the expression of Enam and Odam, but it may not be related to the MTNRs. Basically, melatonin stimulates the differentiation of ALCs, which is consistent with previous experimental results in vivo.

In addition to regulating the expression of the specific enamel genes, melatonin can further manipulate the functional behaviors of ameloblasts. Given that ALCs can calcify spontaneously, melatonin medication will further induce ALCs calcification, especially high concentrations of melatonin (10^−3^ M), as confirmed by microscopic observations of ALP synthesis and mineralized nodule formation. Melatonin can promote ALP activity in ALCs concentration-dependent. Meanwhile, the number of calcified nodules and the positive staining degree of Alizarin Red deepened with increased melatonin concentration. Luzindole, a melatonin receptor inhibitor, significantly inhibited the calcification of ALCs, which suggested that melatonin could regulate the calcification of ameloblasts through Mtnr1a and Mtnr1b.

In conclusion, melatonin reduces the number of ALCs and promotes the differentiation of ALCs. Therefore, it is informative to explore molecular mechanisms of melatonin in the growth and development of ALCs^36^. The Wnt signaling pathway can be divided into the β-catenin-dependent canonical and β-catenin-independent pathways. Previous studies have shown that Wnt/β-catenin signaling pathways are critical for the formation and development of hard tissue, and are involved in various stages of tooth morphogenesis, such as epithelial-mesenchymal interaction, tooth-specific cell proliferation, migration, and differentiation^37^. Various signaling factors in Wnt signaling pathway are expressed in the inner enamel epithelium and ameloblast^38^. Several studies have reported an association between the Wnt signaling pathway and melatonin. For example, melatonin was shown to enhance osteogenic differentiation of BMSCs via the HGF/PTEN/Wnt/β-catenin axis^39^ or H19/miR-541-3p/Wnt/β-catenin axis^40^ and the hypertrophic differentiation of MSC-derived chondrocytes through the Wnt signaling pathway^41^.

Thus, RNA-seq was applied to screen possible signaling pathway, and data showed that genes associated with Wnt signaling pathway changed significantly, indicating the melatonin-medicated activation of Wnt signaling pathway. Interestingly, in our research, the expression of the FZD receptor was significantly increased, and WNT ligand was generally decreased. This finding was consistent with the results of qRT-PCR, in which Wnt4 and Wnt6 were 7- and 11-fold down-regulated, respectively. Scholars believed that the inhibition of Wnt5a family members, including Wnt4, Wnt5a, and Wnt11, effectively activated the canonical pathway^42^. Therefore, we speculated that melatonin activates the canonical pathway by inhibiting the expression of Wnt5a family factors. In the canonical pathway, when the concentration of β-catenin increases, LEF in the nucleus is activated, which then transmits information through downstream target genes Cyclin D1, C-myc and c-Jun^43,44^, to promote the growth and development of the tooth enamel^45^.We then used qRT-PCR to detect the critical signaling molecules involved in the Wnt/β-catenin signaling pathway. Among them, the levels of β-catenin and LEF-1 were significantly upregulated to activate downstream factors. The expression of c-Jun decreased, and Cyclin D1 did not change significantly. These results indicated that Wnt signaling pathway could be significantly activated during melatonin treatment of ALCs. The signaling pathways activated by melatonin in enamel development need to be further explored and verified. At the same time, cell lines cannot simulate the environment in vivo, so future studies will focus on validating these hypotheses using vivo and animal models for pre-clinical trials.

## Conclusion

Melatonin can inhibit the cell number of ALCs and promote differentiation and mineralization. Wnt/β-catenin signaling pathway may be associated with the differentiation of ALCs under melatonin induction. The findings of this study provide an experimental basis for the study of tissue engineering of tooth germ and the factors influencing enamel development abnormality.

## Materials and methods

### Cell culture

ALCs, were obtained from the Ninth People's Hospital Affiliated to Shanghai Jiaotong University School of Medicine (Shanghai, China). The appearance of epithelial cells indicates heterogeneous cell changes in ameloblasts, including differentiation, secretion, transition, and maturation^27^. ALCs were cultured in a standard Dulbecco’s modified Eagle’s medium (DMEM, high glucose, no phosphates, Invitrogen, USA) containing 10% fetal bovine serum (FBS, Invitrogen) and 1% penicillin/streptomycin complex (Invitrogen) at 37 °C in a humidified atmosphere under 5% CO_2_ in the air. The medium was changed twice a week. When the cells reached 70% confluence, they were digested, counted, and transferred to 10 cm culture dishes.

### Cell proliferation assay

ALCs were seeded in 96 well plates at a density of 1 × 10^4^cells/well, and cultured in a standard medium (DMEM as described earlier) at various concentrations of melatonin medium (standard medium containing 10% FBS, supplemented with 10^–10^, 10^–8^, 10^–6^, 10^–4^ and10^−3^ M of melatonin respectively) [Sigma-Aldrich, USA] for 24, 48, 72 and 96 h. The medium was changed every 48 h with the same concentration. At each scheduled time, 10 μl CCK-8 solution (Yeasen, Shanghai, China) was added to each well under dark conditions and then incubated at 37 °C for 2 h. The absorbance at 450 nm was determined using a microplate reader (Tecan, Austria).

### Immunofluorescence

The cell morphology was observed by LSCM. ALCs were seeded into 24 well plates (1 × 10^4^cells/well) and cultured for 48 or 96 h in a standard medium containing melatonin concentrations of 0, 10^–10^, 10^–8^, 10^–6^, 10^–4^, and 10^−3^ M respectively, which were equal to or higher than melatonin circulating levels (10^–10^ to 10^−8^ M)^46^. At the planned time, the samples were fixed in 4% paraformaldehyde, permeabilized with 0.5% Triton X-100, and then stained with rhodamine-labeled phalloidin (1 μg/ml; Sigma Aldrich) and 4,6-diamino-2-phenylindole (DAPI, 1 μg/ml), and finally sealed with Antifade Mounting Medium. LSCM (FV1200; Olympus Company, Japan) was used to observe the samples.

### Quantitative real-time reverse transcription-polymerase chain reaction (qRT-PCR)

qRT-PCR was used to detect the expression of melatonin receptors, such as the Mtnr1a and Mtnr1b, along with specific enamel genes (Amelx, Enam, and Odam). The cells were cultured in a 12-well plate at the density of 1 × 10^5^ cells/well. After 24 h, the medium was removed, and cells were treated with a standard medium containing various concentrations (0, 10^–4^, and 10^−3^ M) of melatonin, and cultured for 1, 2, 3, 4 and 5d. Following the manufacturer’s instructions, total RNA was extracted and isolated with Invitrogen TRIZOL reagents (Thermo Fisher Scientific, Germany). cDNA synthesis was translated from 1 μg total RNA using Hifair II 1st Strand cDNA Synthesis SuperMix (Yeasen). Then, cDNA was amplified in a 20μL reaction mixture using Hieff UNICON qPCR SYBR Green Master Mix (low Rox, Yeasen). The final volume of 20μL contained 10μL of SYBR Green Master Mix (Low Rox), 7.2 μL of DNase/RNase-free water, and 0.2 μM of specific primers. Their sequences are detailed in Table 1. Thermocycling conditions consisted of 95 °C for 5 min, 40 cycles of 95 °C for 10 s and 60 °C for the 20 s each, followed by one cycle at 95 °C for 15 s, 65 °C for 1 min, 95 °C for 15 s, and then stored at 4 °C. The β-Actin gene was used as a housekeeping gene to compare the quantity and quality. Finally, the relative expression of the gene to be tested was determined by the ΔΔCt method.

**Table 1:** Primer sequences used for qRT-PCR.

Genes	GeneBank no	Forward primer sequence (5′–3′) Reverse primer sequence (5′–3′)	Productsize (bp)
Mtnr1a	NM_026642	GAGTCTGTAGTTGTCGCAGTTG GGCCTTTTTGGAAGGAAGAGGA	246
Mtnr1b	NM_205822	GTTATGGCCCTGTTTGTGAGA CAATAGTTCCGGCCTCAAATCAA	125
Amelx	NM_001081978	GATGGCTGCACCACCAAATC CTGAAGGGTGTGACTCGGG	65
Enam	NM_017468	TGCAGAAATCCGACTTCTCCT CATCTGGAATGGCATGGCA	114
Odam	NM_027128	ATCAATTTGGATTCGCACCACC AGTTGGATCTATCCCAGAAGTGA	242
Wnt4	NM_009523	AGACGTGCGAGAAACTCAAAG GGAACTGGTATTGGCACTCCT	126
Wnt6	NM_009526	GCAAGACTGGGGGTTCGAG CCTGACAACCACACTGTAGGAG	202
β-catenin	NM_001081655	GTGTACCCATATCCCAGCCC TCCTGCCCCACATCTCTCAG	239
Lef	NM_172143	ATGGTTAAAGAATACACGGACGC CTGGCAGCCTGGATTTTTGT	163
Cyclin D1	NM_026562	ATGGAAGGACCCTTGAGGC CTTCACGGCTTGCTCGTTCT	123
c-Jun	AJ315350	GTCCTCCATAAATGCCTGTTCC GATGCAACCCACTGACCAGAT	60
β-Actin	NM_027493	ATGACCCAAGCCGAGAAGG CGGCCAAGTCTTAGAGTTGTTG	185

### Quantitative assay of alkaline phosphatase (ALP) activity

The differentiation and mineralization of ALCs were detected by ALP activity. ALCs were seeded into 6-well plates at a density of 1 × 10^5^ cells/well, then cultured in different concentrations of melatonin medium (0, 1 × 10^–4^, 2 × 10^–4^, 5 × 10^–4^, 1 × 10^−3^ M) for 7 days. According to the manufacturer's instructions, the ALP activity of samples was measured with the Alkaline Phosphatase Assay Kit (Beyotime Biotechnology). After cell lysis and extraction, the cells were incubated with 10 μl p-nitrophenol solution (10 mM) for 30 min. In the presence of alkaline phosphatase, the absorbance at 450 nm was monitored by a microplate reader.

### Alizarin red S staining

Alizarin red S staining was used to examine the effects of melatonin and its receptor inhibitor, luzindole (10^−6^ M) [Sigma-Aldrich, USA], on mineralized matrix formation in ALCs. The cells were seeded into 6-well plates at a density of 1 × 10^5^ cells/well for 24 h. Half of the samples were cultured in different concentrations of melatonin medium (the same grouping conditions as the fifth paragraph), and the other half of the samples were exposed to (L-MT−), (L + MT−), (L-MT+), or (L + MT+) for 28 days. Among them, the control group utilized the standard medium (L-MT−), and the three treatment groups involved the standard medium containing luzindole (L + MT−), 10^–3^ M melatonin (L-MT+), or both (L + MT+). After 28 days, the cells were fixed with 4% paraformaldehyde for 30 min and stained with alizarin red solution for 10 min at room temperature. After staining, the cells were cleaned with distilled water and air-dried. Images were taken using an inverted phase-contrast microscope (Leica Microsystems, Germany).

### RNA sequencing and qRT-PCR

Shanghai Majorbio Bio-pharm Technology was responsible for RNA isolation, cDNA library construction, sequencing, and transcriptome data analysis. ALCs were seeded into 60 mm culture dishes at a density of 2 × 10^5^ cells/well, cultured in standard medium or melatonin medium supplemented with 10^-3^ M for 3 days. RNA was extracted with TRIzol. ND-2000 (NanoDrop Technologies) and 2100 Bioanalyzer (Agilent) methods were used to determine the quality of RNA samples, to ensure the use of qualified samples (OD_260/280_ = 1.8–2.2, OD_260/230_ ≥ 2.0, Rin ≥ 6.5, 28S:18S ≥ 1.0, > 2 μg) for transcriptome sequencing library. The RNA library prepared by TruSeq RNA sample preparation Kit from Illumina (San Diego, CA) was sequenced with an Illumina HiSeqxten/NovaSeq 6000 sequencer. The reading length of the sequence was PE 150. The clean reads were then compared with the reference genome using TopHat software (http://tophat.cbcb.umd.edu/, version 2.0.0)^47^. The R statistical package software EdgeR was used for differential expression analysis.

qRT-PCR was performed using the same cDNA of RNA sequencing. The primers used are listed in Table 1.

### Statistical analysis

All experiments were repeated three times, and each experiment was performed in four parallel wells. All data were analyzed using SPSS ver.19.0 (IBM, Armonk, NY, USA) and expressed as mean ± standard deviation (SD). One-way analysis of variance (ANOVA) and Dunn’s multiple comparison test were used to analyze statistical differences. When P < 0.05, the difference was considered statistically significant.

## Data Availability

RNA-Seq datasets have been deposited on Sequence Read Archive (SRA) with accession number: PRJNA 774042.
